# Designing an Optimized Nanocomposite Film Comprising Hydroxypropyl Methylcellulose, Nanoclay, and Dill Essential Oil to Extend the Shelf Life of Eggs

**DOI:** 10.1155/ijfo/6633069

**Published:** 2025-05-15

**Authors:** Mohammad Faghei Shahrbabaki, Alireza Shahab Lavasani, Nazanin Zand, Leila Nateghi, Mohammad Reza Eshaghi

**Affiliations:** Department of Food Science and Technology, VaP.C., Islamic Azad University, Tehran, Iran

**Keywords:** dill essential oil, film, hydroxypropyl methylcellulose, nanoclay, optimization

## Abstract

The use of films in the food industry is very widespread, with a focus on improving the quality of packaging films using nanoparticles and essential oils. The aim of this study was to optimize the formulation of hydroxypropyl methylcellulose (HPMC) film by combining nanoclay and dill essential oil (DEO). The ideal values of nanoclays and essential oils were determined through response surface design and Design Expert software. The results showed that the responses of water vapor permeability and tensile strength of the film were affected by input factors (amount of nanoclay and amount of essential oil), and the final model was estimated to be 4.26 wt% of nanoclay and 1.70 *V*/*w* of DEO. The optimal film was produced and compared with the samples of HPMC film containing nanoclay and the control sample (film without nanoclay and DEO). The optimal sample had the lowest rupture force (428 N), the lowest water vapor permeability (146.91 g.m/m^2^.s.Pa∗10^11^), the lowest solubility in water (24.12%), and the highest antioxidant and antimicrobial activity (75 DPPH) compared to the other samples. In the second stage of the study, raw egg samples were coated with an optimal HPMC film, and their qualitative properties were investigated. It was observed that egg samples coated with optimal layer had the lowest weight loss (4%) and egg white pH (8.7), while the Haugh index (5.56) and egg yolk index (8.3) had the highest value in samples coated with optimal layer. The sensory evaluation of egg samples showed that the optimal samples had the highest score in flavor coefficient (5.3). Based on the results of this study, it can be stated that the use of HPMC film containing nanoclay and DEO has a significant positive effect on improving the quality properties of eggs (*p* < 0.05).

## 1. Introduction

Eggs serve as a valuable source of high-quality protein and essential nutrients. However, the storage of eggs leads to spoilage due to mass transfer involving water vapor and carbon dioxide through the shell, resulting in weight loss and internal quality deterioration, thereby causing economic losses in the poultry industry [1, 2]. Eggs are also subject to internal quality (functional) changes and microbial contamination during storage. With its porous structure, the eggshell allows carbon dioxide and moisture to escape and pollutants such as bacteria and odors to enter the egg. As such, eggs are prone to spoilage and can quickly lose their internal quality, so it is important to protect the shells from mass transfer [2, 3]. Now, in the food industry, various methods are used to disinfect the egg surface, such as dry cleaning or washing with water, which usually contains disinfectants (such as sodium hypochlorite). This method can damage the cuticle, which may contribute to moisture loss and transfer of bacteria through the cuticle [4]. On the other hand, heat pasteurization cannot be used for the whole eggshell because the heat simply cooks the egg. This is why nonthermal processing methods such as electron beams have been proposed as an alternative method to maintain the quality and safety of whole eggshells. However, irradiation under aerobic conditions causes adverse oxidative changes and radiation-induced oxidative changes in whole egg powder and egg yolk solids at more than 3 kGy [3, 4].

To overcome the above problems, considerable attention has been paid to the development of edible coating materials for egg storage, from polysaccharides, proteins, or lipids or their combinations. Edible coating is defined as a harmless (edible) thin layer of food material that forms directly on food surfaces. Therefore, the edible coating is generally recognized as safe (GRAS). Such covers prevent the penetration of microorganisms into the eggshell. As a result, they increase their storage time and reduce economic losses. Previous studies have concluded that coatings help maintain internal quality, increase shell strength, and reduce microbial load on the surface of the shell [4–6]. These biodegradable coverings can accommodate various additives, including antioxidant, antimicrobial, and antifogging agents, and offer several advantages over petroleum-based plastics, such as greater availability, reduced costs, and simplified operations. These properties enhance the functional aspects of food, extend shelf life, and yield healthier food products [7].

Hydroxypropyl methylcellulose (HPMC), a cellulose derivative obtained through propylene oxide and methyl chloride reaction, remains a nonionic polymer and forms a highly stable solution within a pH range of 3–11. It demonstrates resistance to high temperatures (between 120°C and 190°C) when appropriately combined with plasticizers like glycerol or sorbitol. Approved by the Food and Drug Administration for nontoxicity and edibility, HPMC stands as a suitable polymer for film formation. Previous research has indicated that this polymer can produce a film with adequate strength, transparency, odorlessness, oil penetration resistance, and water solubility [8, 9]. HPMC exhibits lower water vapor permeability (WVP) than protein films, making it a favorable candidate for film and covering applications, particularly in scenarios requiring controlled water transfer through eggshells [10]. Essential oils from various plants possess significant antioxidant and antimicrobial properties attributed to hydrogen-containing and functional groups within their chemical structure [11].

“Dill (*Anethum graveolens*),” an annual plant belonging to the Umbelliferae family, serves culinary and medicinal purposes in numerous countries. The essential oil extracted from dill's leaves and seeds contains diverse compounds such as carvone, limonene, phellandrene, eugenol, and eucalyptol, harboring substantial antioxidant and antimicrobial activities [12]. Biopolymers employed for covering applications, including HPMC, often exhibit inadequate strength, necessitating enhancement. Incorporating a low percentage of nanoclay into polymers has been shown to improve their mechanical properties and thermal stability. Furthermore, the addition of nanoclay results in the retardation of molecular diffusion, thereby enhancing moisture, oxygen, carbon dioxide, and UV barrier properties in comparison to conventional composites. A specific type of nanoclay, montmorillonite (MMT), is commonly utilized to fabricate nanocomposite films and coverings owing to its cost-effectiveness, nontoxic nature, and widespread availability [13–15].

This study is aimed at optimizing the HPMC film production process by integrating nanoclay and dill essential oil (DEO). The resulting covering formulation will be utilized to envelop eggs, and its impact on shelf life and quality will be thoroughly evaluated in subsequent stages.

## 2. Materials and Methods

### 2.1. Materials

Eggs were purchased from a local supplier (Tehran, Iran). DEO was purchased from Barij Essence Pharmaceutical Co. (Kashan, Iran) and stored in a dark container at 4°C until used. HPMC powder composed of 9% hydroxypropyl and 28% methyl radicals with water solubility (2%) at 25°C and a viscosity of 15 MPa s was provided by Sigma Chemical Co. (Oakville, Ontario, Canada). Nanoclay (sodium MMT) was purchased from the Iranian Nanomaterials Pioneers Company (Mashhad, Iran). 2,2-Diphenyl-1-picrylhydrazyl (DPPH), glycerol, Mueller–Hinton agar (MHA), and Mueller–Hinton broth (MHB) were obtained from Merck Co. (Darmstadt, Germany). The studied bacteria, including *Escherichia coli* ATCC 25922 and *Staphylococcus aureus* ATCC 25923, were provided by the Iranian Research Organization for Science and Technology (Tehran, Iran).

### 2.2. Methods

#### 2.2.1. Film Preparation

The film was created following the procedure outlined in Reference [16], with a few adjustments. First, 4 g of HPMC powder were combined with 50 mL of distilled water and left to mix for 2 h at a temperature of 65°C and a speed of 1200 rpm. After this, 2 g of glycerol were introduced to the solution. In the subsequent step, precise quantities of nanoclay and DEO, determined based on the findings from the response surface design (4.26 wt% nanoclay and 1.70%*v*/*v* DEO), were added to the HPMC film-forming solution. The final mixture was then heated to 24°C and maintained at this temperature for 18 min. Film solutions were poured on plates with a diameter of 15 cm and transferred into an oven for complete drying (35°C for 30 h). In the last step, the dried films were peeled from the surface of the plates and stored at ambient temperature for further analysis. Treatments are prepared as Table 1.

#### 2.2.2. Film Properties

##### 2.2.2.1. Mechanical Characterization

Tensile strength (TS) and elongation at break (EB) were evaluated by the ASTM standard method D882 [17] using a texture analyzer TA-XT-PLUS (Stable Micro Systems Co. Ltd., England) at 25°C to assess the tensile properties of preconditioned films. Before conducting the tests, five rectangular strips measuring 10 × 1.5 cm were extracted from each film sample. The initial cross-head speed and grip spacing were set at 50 mm/min and 50 mm, respectively. Subsequently, the TS and EB values were calculated using the specified equations (Equations (1) and (2)):

(1) $$\begin{array}{c} \text{TS }\left(\text{MPa}\right)=\frac{\text{Maximum Force}}{\left(\text{Film width}\right)\times \left(\text{Film thickness}\right)}, \end{array}$$

(2) $$\begin{array}{c} \text{EB }\left(\%\right)=\frac{\left(\text{Maximum length before breaking}\right)\times \left(\text{Initial length}\right)}{\left(\text{Initial length}\right)}\times 100. \end{array}$$

##### 2.2.2.2. WVP

The WVP of the film samples was determined using a gravimetric method at ambient temperature and 75% relative humidity, according to the ASTM standard [18]. Each cup, with a surface area of 0.00287 m^2^, contained approximately 100 g of anhydrous calcium chloride (0% RH, assay cup) and was sealed with film samples in triplicate. These cups were then placed into desiccators containing a saturated sodium chloride solution at 75% RH. The difference in relative humidity between the inner and outer atmosphere of the cups corresponded to a partial pressure of water vapor of 1753.55 Pa. The weight gain of the cups was measured every hour for 12 h with an accuracy of 0.0001 g. The WVP and water vapor transmission rate (WVTR) were then calculated based on the data collected (Equations (3) and (4)):

(3) $$\begin{array}{c} \text{WVTR}=\frac{\text{The weight gain versus time slope }\left(\text{g}/\text{s}\right)}{\text{Exposed surface area of film }\left({\text{m}}^{2}\right)}, \end{array}$$

(4) $$\begin{array}{c} \text{WVP}=\text{WVTR}\times \frac{\text{Film thickness }\left(\text{m}\right)}{\text{Difference of pressure between the inner and outer sides of cup }\left(\text{Pa}\right)}. \end{array}$$

##### 2.2.2.3. Structural Characterization

A Fourier transform infrared (FTIR) spectrometer (370 Avatar, United States) was utilized to examine the chemical bonds present in the prepared nanocomposite films. The ATR-FTIR spectra of the nanocomposites were acquired within the 450–4000 cm^−1^ range at a resolution of 4 cm^−1^. Each sample underwent an average of 16 scans, and all measurements were conducted at ambient temperature [19].

##### 2.2.2.4. Antibacterial Properties

The antibacterial activity of the films was evaluated using the agar disc diffusion method. Initially, 1 mL of a bacterial suspension containing 10^8^ CFU/mL was aseptically inoculated onto MHA in Petri dishes. Subsequently, the films were meticulously cut into 6 mm discs and aseptically placed onto the Petri dishes. Following a precise incubation period of 24 h at 37°C, the diameter of the inhibition zones was accurately measured. Each film underwent three replications, and the average measured value was diligently recorded and reported [16].

##### 2.2.2.5. Antioxidant Properties

The determination of DPPH radical scavenging activity follows the protocol established by Brand-Williams et al. [20], as referenced by Yekta et al. and Shojaee-Aliabadi et al. [19, 21]. Briefly, 25 mg of each treatment is dissolved in 5 mL of distilled water and then mixed with 3.9 mL of DPPH methanol solution. Subsequently, the resulting mixture is left in a dark environment for 1 h at room temperature. Following this incubation period, the absorbance (A) is measured at 517 nm against a blank sample. The DPPH radical scavenging activity (%) is then calculated using Equation (5):

(5) $$\begin{array}{c} \text{DPPH scavaging activity }\left(\%\right)=\frac{\left(\text{A blank}\right)-\left(\text{A treatment}\right)}{\text{A blank}}\times 100. \end{array}$$

##### 2.2.2.6. Scanning Electron Microscopy (SEM)

The microstructure of the produced films was investigated by Tescan Vegan-3 model JDM-35 microscopy made in the United Kingdom. To investigate the effect of adding nanoclay and DEO on the microstructures of the produced film, electron microscopy images were prepared from the surface of the films. First, the films were glued to the aluminum base with the help of silver glue. The piers were dried to the critical point in a coating/spraying machine and coated with gold for 5 min. Imaging of the samples was done by SEM with a 20 kW magnification of 10,000 times and a resolution of 2 mm [19].

##### 2.2.2.7. X-Ray Diffraction (XRD) Spectrometer

For analysis, each film sample is first made into a very fine powder with a weight of 0.5–3 g and exposed to the bombardment of x-ray rays at a wavelength of 0.1–100 Å. The result is a diffraction pattern. Each crystal specimen has a unique diffraction pattern that can be compared with standard diffraction patterns; the compound is identified [18].

##### 2.2.2.8. Studying the Release of Essential Oil From the Film

Release of essential oil from film samples in 95% ethanol (food simulator) was evaluated. Briefly, the film samples were cut into 2 × 2 cm^2^ and immersed in vials containing 10 mL of simulator. The vials were then stored in the dark at different temperatures (4°C and 25°C) for 72 h with shaking twice daily. Finally, the amounts of essential oil released were measured using a UV–vis spectrophotometer at 329 nm [19].

#### 2.2.3. Covering Eggs With Optimized Film

After washing and drying fresh eggs with warm water, all samples were immersed in the solutions for approximately 15 min and stored at room temperature. Their quality properties, including weight loss, HU indices, yolk index, and egg white pH, were measured weekly for 6 weeks [22].

#### 2.2.4. Characterization of the Eggs During Shelf Life

##### 2.2.4.1. Weight Loss

Following the surface covering and drying process, the samples' weight loss was quantified using the prescribed Equation (6) [22].

(6) $$\begin{array}{c} \text{Weight loss }\left(\%\right)=\frac{\left(\text{Initial weight of the egg}\right)-\left(\text{Weight of the egg after a certain period of storage}\right)}{\text{Initial weight of the egg}}\times 100. \end{array}$$

##### 2.2.4.2. HU Indices

The egg indices were determined by measuring the height of the albumen using a digital micrometer (Mitutoyo 293 model). The following equation used the obtained value to calculate the HU index (Equation (7)) [13].

(7) $$\begin{array}{c} \text{HU Index}={\text{Log}}^{\left(H-1.7\;W^{0.37}+6.7\right)}, \end{array}$$

where *W* is the egg weight (grams) and *H* is the height of the albumin (millimeters).

##### 2.2.4.3. Yolk Index

In order to determine the yolk index, a digital micrometer (Mitutoyo 293 model, Japan) was utilized to measure the height and width of the yolk, and the resulting values were incorporated into the following equation (Equation (8)) [23].

(8) $$\begin{array}{c} \text{Yolk index }\left(\%\right)=\frac{\text{Height of the yolk}}{\text{Width of the yolk}}\times 100. \end{array}$$

##### 2.2.4.4. Egg White pH

The egg yolks were separated from the egg whites to measure the pH of the egg whites. The pH was determined using an electric pH meter (Metrohm, Switzerland).

#### 2.2.5. Experimental Design and Statistical Analysis

##### 2.2.5.1. Response Surface Methodology (RSM)

Design Expert software (Version 13, MN, United States) was used to investigate statistical analysis and regression modeling. A central composite design at three levels for each independent variable at three center points replicates was performed for the research. Thirteen different edible film formulations, including DEO (0–2 vol%/vol%) and nanoclay (0–5 wt%), were defined to get the best optimal condition (Table 1). The effect of DEO and nanoclay concentrations (independent variables, *X*) on the resulting film properties (response functions, *Y*), including WVP, TS, and EB, was evaluated. The complete design matrix of the experiments is demonstrated in Table 1. The experimental sequence was randomized to minimize the effects of the uncontrolled factors. A second-order equation was used to fit the data as a function of dependent variables (Equation (9)):

(9) $$\begin{array}{c} Y_{n}=\beta_{0}+\beta_{1}X_{1}+\beta_{2}X_{2}+\beta_{11}X_{1}^{2}+\beta_{22}X_{2}^{2}+\beta_{12}X_{1}X_{2}+\epsilon , \end{array}$$

where *Y*_*n*_ is the predicted response, *X*_1_ and *X*_2_ are independent variables, *β*_0_ is the constant coefficient, *β*_1_ and *β*_2_ are the linear coefficients, *β*_12_ is the interaction coefficient, and *β*_11_ and *β*_22_ represent the quadratic coefficients. Finally, *ε* signifies the error related to the predicted response (Table 2).

The independent variables and their code variable levels are shown in Table 3. The model equations were developed using the Design Expert software, 2D contour plots of the responses were graphed, and the optimum conditions of the independent variables were predicted. The two independent variables were *X*_1_ (nanoclay concentration %) and *X*_2_ (DEO concentration %).

#### 2.2.6. Verification, Optimization, and Statistical Analyses

Design Expert software (Version 13, MN, United States) was utilized to predict the optimized blend formula of the independent variables for covering eggs. Analysis of variance (ANOVA), the coefficient of determination (*R*^2^), and the adjusted coefficient of determination (*R*^2^_Adjusted_) were used to evaluate the model's validity. The optimum level of two independent variables (*X*_1_: nanoclay and *X*_2_: DEO) was predicted using the numerical and graphical optimization measures to obtain the best film properties, including TS (megapascal), EB (percent), and WVP (g.s^−1^.m^−1^.Pa^−1^). The results represent the mean with standard deviation (±SD) from three trials for each test. ANOVA was employed to ascertain the significance level of the models using SPSS software (Version 25, IBM, United States). The *p* value and lack of fit (LOF) of the selected models should be < 0.05 and > 0.05, respectively.

## 3. Results and Discussion

### 3.1. Fitting of the Model

In this study, the RSM was used to optimize the formulation of HPMC film with two variables, nanoclay and DEO concentrations, for egg covering.

Table 2 exhibits the experimental results attained by the response variables. A regression analysis was applied to fit a full response surface model for all examined responses, comprising linear (*X*_1_ and *X*_2_), interaction (*X*_1_*X*_2_), and quadratic terms (*X*_1_^2^ and *X*_2_^2^).

The impact of independent variables, that is, nanoclay and DEO, in the film, was analyzed and evaluated for their effects on WVP, TS, and EB. The results are presented in Table 3. The results obtained in Table 3 were analyzed using a second-order model. An ANOVA was performed to estimate the significant effects and interactions of the independent variables. A multivariable correlation analysis was also conducted to obtain the coefficients of the final equation. Variables that did not significantly affect the response were removed to get the final model equation. The model's performance was evaluated by examining the LOF, *R*^2^ coefficients, Adj‐*R*^2^, and *p* values.

Table 4 illustrates that all attained models were significant (*p* < 0.01). In the case of WVP and EB, the quadratic model was significant (*p* < 0.01). Moreover, the linear model was significant (*p* < 0.01) for TS. Additionally, the CV for all the screening parameters was less than 10%, an acceptable value for the modeling (Table 4).

In the current investigation, the coefficient of determination (*R*^2^) values for WVP, EB, and TS were 0.939, 0.855, and 0.966, whereas the Adj‐*R*^2^ values were 0.896, 0.826, and 0.943, respectively. Consequently, the dependent variables were found to have significant quadratic models (*p* < 0.05).

The results showed that the optimal amounts of nanoclay and DEO in the film formulation were 3% and 1.25%, respectively. As the nanoclay concentration in the formulation increased, the film resistance also increased, while as the DEO concentration increased, the film resistance decreased. Moreover, as the concentration of DEO and nanoclay in the formulation increased, the water vapor transfer rate from the film decreased.

### 3.2. Influence of Main and Interaction Effects on Responses

#### 3.2.1. WVP

**Figure 1:**
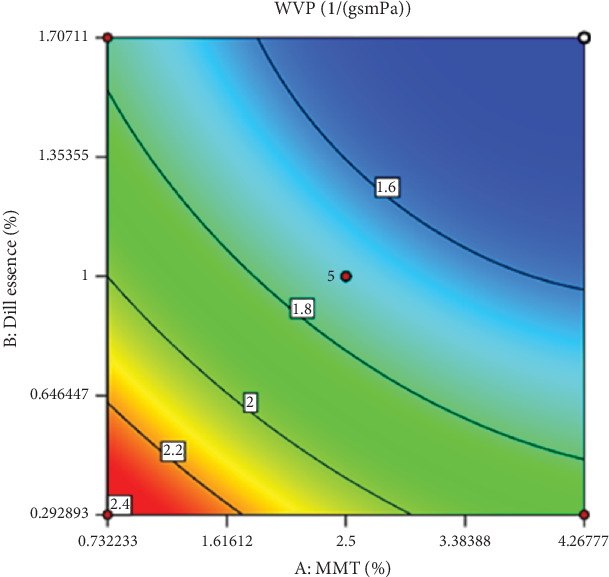
The effect of nanoclay (MMT) and DEO (dill essence) on the changes in WVP (g.s^−1^.m^−1^.Pa^−1^).

Considering the importance of nanoclay in producing polymer films, finding the most effective amount of nanoclay is crucial. As shown in Figure 1, the lowest WVP was related to the highest amounts of nanoclay and DEO. According to Table 3, the effect of nanoclay and DEO and their interaction was significant at a 95% confidence level (*p* ≤ 0.05), with more substantial changes shown in Figure 1.

Based on Table 4, the presented model for WVP analysis was a second-order model, which was significant at a 95% confidence level. The correlation coefficients *R*^2^, Adj‐*R*^2^, and *R*^2^_Pre_ also showed acceptable values. The nonsignificant value of the LOF indicated that the presented model is sufficient for predicting the efficiency of WVP. After removing the nonsignificant values from the model, the presented model for WVP analysis could be expressed as follows:

 $$\begin{array}{c} \text{WVP}=25.18-0.2X_{1}-0.26X_{2}+0.06X_{1}X_{2}+0.08X_{1}^{2}+0.09X_{2}^{2}. \end{array}$$

It has been reported that based on the fitted coefficients (Table 5), the proposed models have high adequacy and can be used to predict WVP. Nanoclay and DEO prevent the interaction between water molecules and OH groups in polymer chains and reduce the moisture content. Nanoclay and DEO can also fill cavities and voids inside the film and prevent water molecules from settling inside the cavities. The decrease in permeability to water vapor is attributed to the presence of nanoclay particles with a high aspect ratio and the creation of a long zigzag path for the penetration of water vapor molecules. The more and better the nanoclay layers are spread in the biopolymer matrix, the more these zigzag paths increase and the film's resistance to water vapor and gases increases. The use of nanoparticles to reduce WVP in various films and coverings has been widely studied. In a similar study, Bi et al. [24] investigated the use of silica nanoparticles to minimize WVP in plastic films and demonstrated that the incorporation of silica nanoparticles in plastic films led to a reduction in WVP. These nanoparticles acted as a reinforcing and filling agent in films and improved water vapor barrier properties [24]. The decrease in WVP in the presence of nanoparticles has been attributed to their high aspect ratio and the creation of long and tortuous paths for the diffusion of water vapor molecules [19, 25, 26]. The more the nanoparticle layers are dispersed and distributed well in the biopolymer matrix, the more these tortuous paths are increased, and the film's barrier properties against water vapor and gases are enhanced [27].

WVP experienced a declining trend with increasing DEO concentration up to 1.7% vol/vol. A hydrophobic disperse phase restricted water vapor diffusion through the polymeric matrix and augmented the tortuosity factor of mass transfer (Table 5) [28]. However, it was reported that the excess amount of essential oils could disrupt the interactions in the polymeric matrix of films, creating some irregularities and voids and increasing WVP. The lowest WVP value (1.49 × g.s^−1^.m^−1^.Pa^−1^) was obtained in the treatment containing 4.26 wt% nanoclay and 1.70% vol/vol DEO.

#### 3.2.2. TS

**Figure 2:**
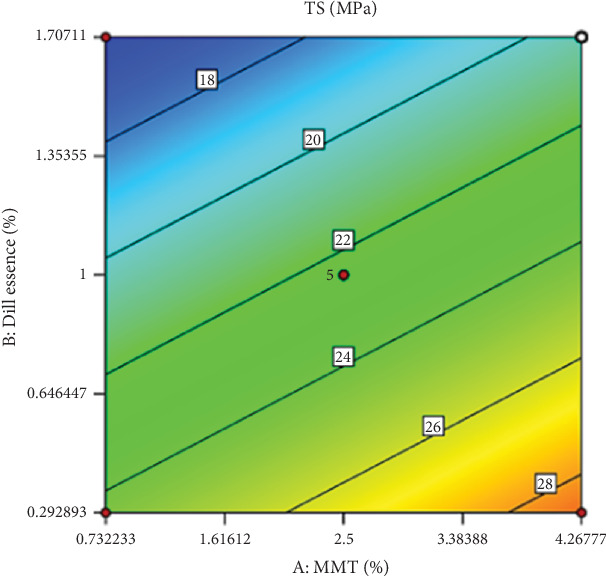
The effect of nanoclay (MMT) and DEO (dill essence) on the changes in the tensile strength (MPa) factor.

The investigation the interaction effect between the independent variables has shown a significant impact on the model for the interaction between the amount of nanoparticles and the amount of DEO (*p* ≤ 0.05). The TS value of HPMC-based films was enhanced by increasing nanoclay dosage at all concentrations of DEO (Figure 2). A number of mechanisms were suggested about the latest phenomenon, including the improvement of cross-linking between polymeric chains as a result of the interaction between nanofiller and polymer in the matric, the filling of empty voids and small cracks in amorphous regions of polymer, declining the flexibility and mobility of polymer chains, and improving the film's crystalline structure due to the high crystallinity of nanofillers.

On the other hand, increasing the dosage of DEO concentration led to a decline in TS. HPMC films were primarily stabilized by weak interactions, including hydrophobic interactions as well as hydrogen bonds. Weaker polymer–oil interactions could substitute the stronger polymer–polymer interactions in the matrix due to incorporating DEO into the film-forming solution, weakening the polymer network cohesiveness and eventually diminishing the TS of the film.

**Figure 3:**
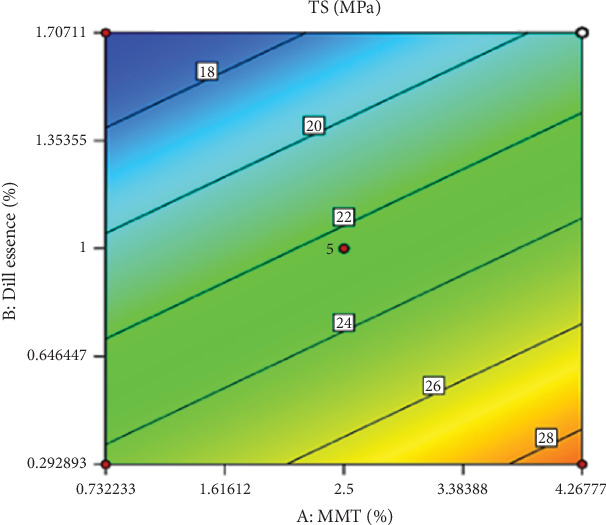
The effect of nanoclay (MMT) and DEO (dill essence) on the changes in the tensile strength (MPa) factor.

The amount of DEO (*p* < 0.05) was significant. The TS value of HPMC-based films was enhanced by increasing nanoclay dosage at all concentrations of DEO (Figure 3). A number of mechanisms were suggested about the latest phenomenon, including the improvement of cross-linking between polymeric chains as a result of the interaction between nanofiller and polymer in the matric, the filling of empty voids and small cracks in amorphous regions of polymer, declining the flexibility and mobility of polymer chains, and improving the film's crystalline structure due to the high crystallinity of nanofillers. On the other hand, increasing the dosage of DEO concentration led to a decline in TS. Weak interactions, including hydrophobic and hydrogen bonds, primarily stabilize HPMC films. Weaker polymer–oil interactions could substitute the stronger polymer–polymer interactions in the matrix due to incorporating DEO into the film-forming solution, weakening the polymer network cohesiveness and eventually diminishing the TS of the film.

After examining the fitted coefficients (Table 4), it was revealed that the values of *R*^2^, Adj‐*R*^2^, and *R*^2^_Pred_ coefficients were high and acceptable, which confirms the validity of the proposed model. Additionally, the LOF value indicates the insignificance of the model and its high adequacy. By removing the noneffective variables from the model, the final model was expressed as a coded linear equation as follows:

 $$\begin{array}{c} \text{TS}=22.43+2.14A-4.9B. \end{array}$$

#### 3.2.3. EB

Based on Table 3, the fitting coefficients (Table 4), and the estimated LOF, the results indicate the model's high efficiency and adequacy in evaluating the EB level. By removing the insignificant variables, the coded model was expressed as follows:

 $$\begin{array}{c} \text{EB}=24.72-2.73A+4.19B+1.15\text{AB}+0.42A^{2}-1.49B^{2}. \end{array}$$

TS and EB in HPMC-based films are primarily attributed to hydrogen bonds between the HPMC chains. These bonds contribute to low cohesiveness and flexibility in nonplastic films [29]. Additionally, the increase in essential oil content increases the EB of all treatments, as oil acts as a stabilizer and increases the film's stability [30].

Increasing the nanoclay level can reduce the film's EB, decreasing the film's percentage elongation. Xu et al. [31] reported on the properties of a nanocomposite film, indicating that with a slight increase in the amount of nanoresin, the percentage elongation in the chitosan–nanoresin polymer matrix decreased.

**Figure 4:**
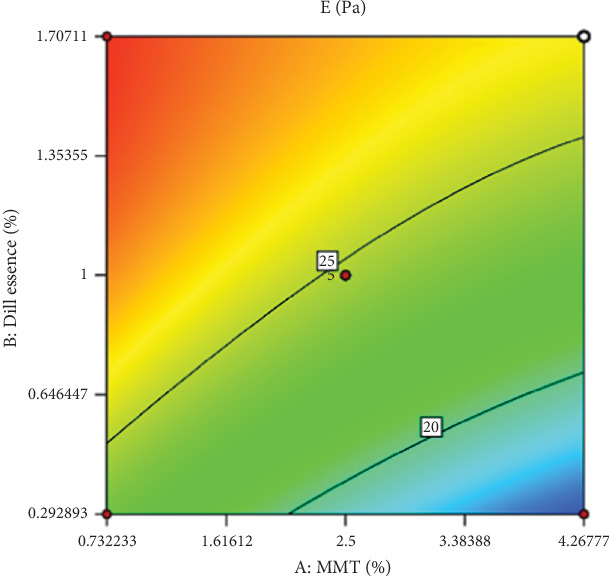
The effect of nanoclay (MMT) and DEO (dill essence) on changes in EB (percent).

The proposed optimal formula's desired objective and optimization criterion is a nanoclay content of 4.26 wt% and a DEO content of 1.70% vol/vol. Under these conditions, a WVP of 1.54 g.s^−1^.m^−1^.Pa^−1^, a predicted TS of 23.55 MPa, and an EB of 23.64% are expected (Figure 4). The optimal formulation conditions were tested in three repetitions, and the results confirmed no significant difference in WVP, TS, and EB.

Radfar et al. [16] used nanoresin and date seed extract as optimization factors for producing nanocomposite films. The results showed that adding nanoresin and extract to the film solution improved the film's mechanical properties and moisture sensitivity, which is similar to the results of this study. In another study, researchers investigated the effect of adding nanoclay and fennel essential oil on the properties of HPMC films [32]. The results showed that adding 0.5% weight of nanoclay and essential oil improved the film's mechanical properties and moisture sensitivity, which is consistent with the results of this study. Malik and Mitra [33] examined the effect of zinc nanoparticles on the properties of HPMC films. The results showed that adding zinc nanoparticles to the HPMC solution improved the film's mechanical properties and moisture sensitivity. Furthermore, the microbial and antioxidant properties and FTIR analysis were investigated among the optimal films containing nanoresin and those without it.

### 3.3. Antimicrobial and Antioxidant Activity Evaluations

**Figure 5:**
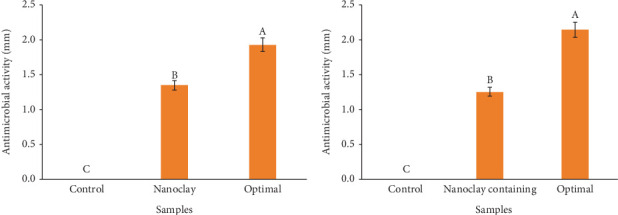
Antimicrobial activity of treatments: control: HPMC film, nanoclay: HPMC-based film containing 5 wt% nanoclay, and optimal: HPMC film containing 4.26 wt% nanoclay and 1.70% vol/vol DEO against (a) *S. aureus* and (b) *E. coli*.

In Figure 5a, it is shown that the optimal sample (4.26% weight nanoclay and 1.70% volume DEO) had higher antimicrobial activity against *S. aureus* compared to other samples (control and sample containing nanoclay). The difference in antimicrobial activity between the optimal sample and other samples was significant at a 95% confidence level (*p* ≤ 0.05).

Figure 5b shows the changes in antimicrobial activity of the optimal film sample, control, and nanoclay-containing sample against *E. coli* bacteria. According to the presented results, the optimal sample had the highest antimicrobial activity against *E. coli* compared to the control and nanoclay-containing samples (*p* ≤ 0.05).

**Figure 6:**
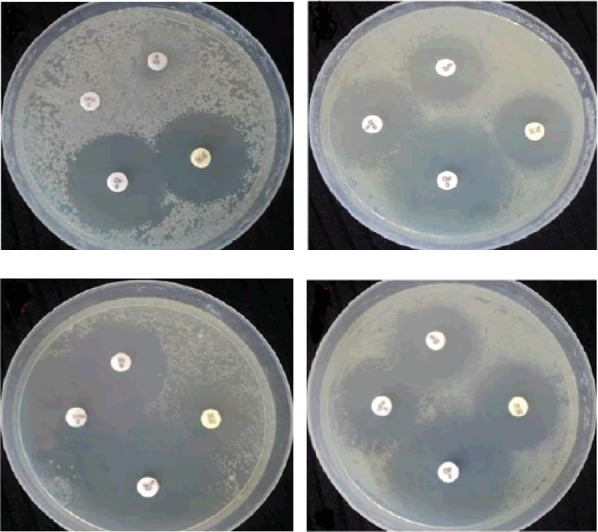
Photos of the microbial inhibition halo plates for the mentioned bacteria. (a) Film containing silver nanoparticles, (b) film containing basil leaf essential oil, (c) films containing silver nanoparticles and basil leaf essential oil, and (d) films containing silver nanoparticles and basil leaf essential oil.

Nanocomposite films contain nanoscale particles and active nanomaterials that can improve polymeric films' physical and chemical properties. Nanoparticles and essential oils have been used in a HPMC film, which has added antimicrobial properties. In an investigation, it has been shown that the use of silver nanoparticles and basil leaf essential oil in HPMC films has positively affected the film's antimicrobial properties. The results have shown that films containing silver nanoparticles and basil leaf essential oil have significantly improved antimicrobial properties compared to films containing only one of these materials, which is consistent with the results of this study (Figure 6) [34].

**Figure 7:**
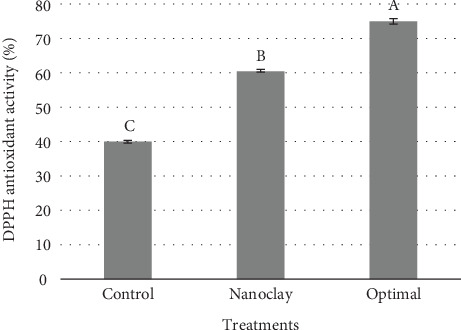
Antioxidant activity of treatments: control: HPMC film, nanoclay: HPMC-based film containing 5 wt% nanoclay, and optimal: HPMC film containing 4.26 wt% nanoclay and 1.70% vol/vol DEO. Different letters on each bar indicate significant differences at *p* < 0.05.

Nanoparticles can increase the antioxidant activity by increasing the surface area and the surface-to-volume ratio. For example, silver nanoparticles, due to having more surface than volume, can actively interact with free radicals and a chemical oxidation that occurs during biological and chemical processes in biological systems, interacts and, by reducing them, performs antioxidant activity. In addition, some nanoparticles, such as titanium dioxide nanoparticles and clay nanoparticles with compounds such as rutin, quercetin, and vitamins C and E, have been combined with these compounds that have antioxidant properties and play an important role in the antioxidant activity of nanoparticles. In general, nanoparticles can increase the antioxidant activity due to their greater surface area and ability to interact with free radicals, and as a result, they can help improve the antioxidant properties of polymer films. The results presented in Figure 7 demonstrate that the optimal sample (4.26 wt% of nanoclay and 1.70% vol of essential oil) had the highest ability to scavenge DPPH free radicals, which was statistically significantly different from the other samples (*p* ≤ 0.05). Scientific studies have shown that the use of nanoparticles and essential oils in the structure of HPMC films can increase their antioxidant activity. One of the studies that has addressed this issue is the study by Shankar et al. [35]. As can be seen, films containing silver nanoparticles and plant essential oils such as citrus and cinnamon essential oils have high antioxidant activity. The results showed that the antioxidant activity of films containing silver nanoparticles and essential oils is higher than that of those containing only one [35]. Therefore, using nanoclay and essential oils in the structure of HPMC films can increase their antioxidant activity. Additionally, some compounds in essential oils, such as terpenoids and phenols, have antioxidant properties that can significantly improve the antioxidant activity of the films [36].

### 3.4. FTIR

FTIR spectroscopy is a technique used to analyze the chemical composition of materials. It provides information about functional groups, chemical bonds, and possible chemical interactions among different components of a material.

**Figure 8:**
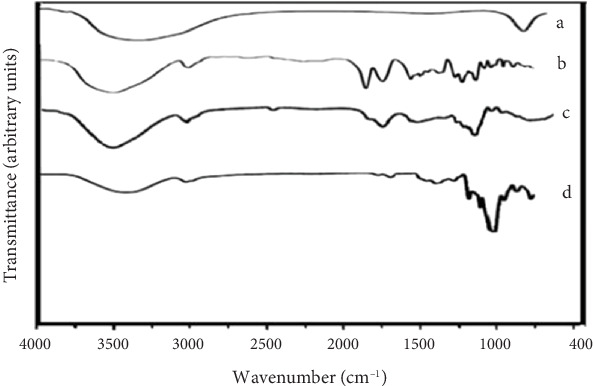
FTIR spectra of treatments of (a) essential oil, (b) HPMC, (c) HPMC-based film containing 5 wt% nanoclay, and (d) HPMC film containing 4.26 wt% nanoclay and 1.70% vol/vol DEO.

Generally, the following peaks were observed in the pure HPMC film )control(.  Figure 8 shows a broad peak between 3000 and 3600 cm^−1^, corresponding to the intermolecular hydrogen bonding of hydroxyl groups in the film samples. This peak appeared in all three samples and could be attributed to either the water in the film matrix due to the presence of plasticizer or free hydroxyl groups in the polymeric structure of HPMC. The symmetric stretching vibration of C–H bonds in methyl (CH_3_ or CH_2_) groups present in the structure of hydroxypropyl cellulose occurs at around 2820–2888 cm^−1^. The band observed at 1024 cm^−1^ is likely to be C–O stretching vibration [23, 37]. A small but distinct peak at around 1710 cm^−1^ was observed in all spectra, corresponding to carbonyl (C=O) groups in the hydroxypropyl cellulose units [38]. At 1397 cm^−1^, a sharp peak due to C–O–C and symmetric bending vibration of methoxy groups was observed, related to the presence of cyclic ether in the HPMC structure. On the other hand, a relatively small peak in the 1000–1100 cm^−1^ range was observed, corresponding to the stretching vibration of the C–O–C group. Another small peak at around 930 cm^−1^ was also observed, corresponding to the asymmetric stretching vibration of the pyranose ring in the HPMC structure. In the FTIR spectrum of the HPMC film, peaks in regions 1018 and 1160 cm^−1^ show the C–O tensile vibrations of the carboxylic acid and ester groups. The 2001 peak confirms the vibrations of the –CH3 groups. The 1450 peak shows C–C vibrations within aromatic rings. The peak in region 2990 cm^−1^ shows the vibrations of RCOOH and C–C–CO–OH. According to the mentioned cases, most of the functional groups in the structure of HPMC are visible in this spectrum. In the FTIR spectrum of nanoclay, the peak in Region 610 shows the tensile vibrations of clay, and the peak in Region 3300 probably corresponds to the vibration of OH groups of possible water molecules [23]. In the FTIR spectrum of HPMC film containing 4.26 wt% nanoclay and 1.70% vol/vol DEO films, almost all peaks related to HPMC, nanoclay, and DEO are observed, but some of these peaks have shifted to different wavelengths indicating electrostatic interactions between HPMC, nanoclay, and DEO.

### 3.5. Thermal Analysis

**Figure 9:**
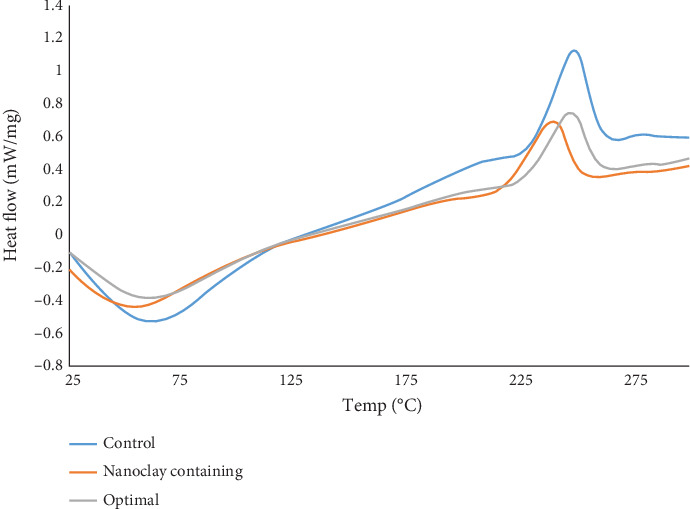
DSC graphs of treatments: control: HPMC film, nanoclay: HPMC-based film containing 5 wt% nanoclay, and optimal: HPMC film containing 4.26 wt% nanoclay and 1.70% vol/vol DEO.

Thermograms of films showed a small shoulder between these two peaks, which corresponds to the glass transition temperature (*T*_*g*_) of the samples (Figure 9). *T*_*g*_ is the temperature region where the polymer transforms from a hard, glassy material to a soft, rubbery material. The lowest *T*_*g*_ was observed in the control sample, which was 148.8°C. With the addition of nanoclay, its value increased to 166.1°C, and with the addition of essential oil, it increased to 162°C. The increase in *T*_*g*_ values of the samples compared to the control film may be due to the slower mobility of the polymer chains, possibly due to their stronger interaction with the added nanorice or essential oil. In other words, in HPMC films containing nanoclay, the nanoclay can create a strong interaction with the polymer chains and limit the mobility of the surrounding chains [39].

This state is similar to the formation of a crosslink between these two compounds. On the other hand, when nanoclay is dispersed in the polymer matrix, a new nanorice may be formed, which reduces the mobility of many polymer chains. Structurally, it can also be said that films containing nanoclay behave like a semicrystalline polymer, as nanoclay behaves like crystalline particles [40]. These results are similar to what Otoni et al. [41] reported about the glass transition temperature of HPMC films with different degrees of substitution. Additionally, Lorevice et al. [42] reported that the glass transition temperature of the film matrix increases with the addition of nanoclay, which is consistent with our results.

### 3.6. Study of SEM Images

**Figure 10:**
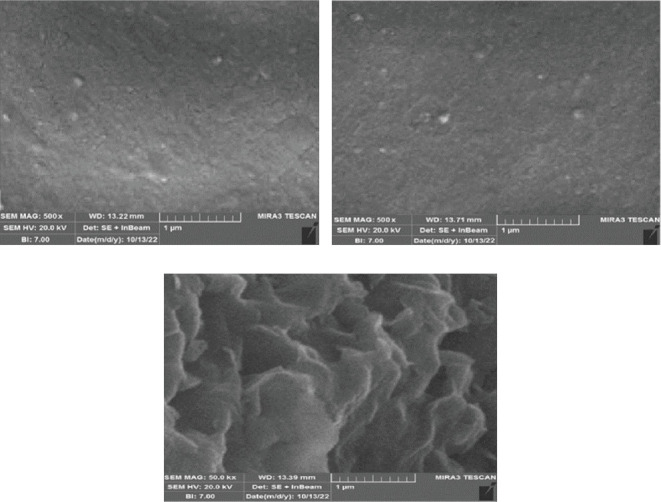
SEM images of treatments. (a) HPMC film, (b) HPMC-based film containing 5 wt% nanoclay, and (c) HMPC film containing 4.26 wt% nanoclay and 1.70% vol/vol DEO.

Figure 10 shows the SEM images of the HPMC film and its composites. According to the SEM images, the film shows a smooth, uniform, and somewhat cavity-free surface. However, by adding encapsulated essential oil to the film (HPMC-based film containing 5 wt% nanoclay), the film surface becomes more uneven, and pores are created on the film surface. This result confirms the results of WVP because the film with the highest percentage of encapsulated essential oil has shown the highest WVP, which has also increased the WVP due to the pores created in the film. The film contains nanoclay and DEO nanoparticles (HPMC film containing 4.26 wt% nanoclay and 1.70% vol/vol DEO) showing an almost nonsmooth and rough surface, which is probably due to the presence of nanoparticles on the film surface. In a film in which both nanoclay and DEO nanoparticles and encapsulated essential oil are present at the same time, the surface of the film has the most nonuniformity and the most roughness, and in this film, the most porous pores are observed. Tripathi et al. [43] investigated the structural morphology of the chitosan/pectin film. They report that the pure pectin film and the chitosan/pectin composite have a smooth surface with almost no cavities, which is consistent with the results of the present study.

### 3.7. Study of Crystalline

**Figure 11:**
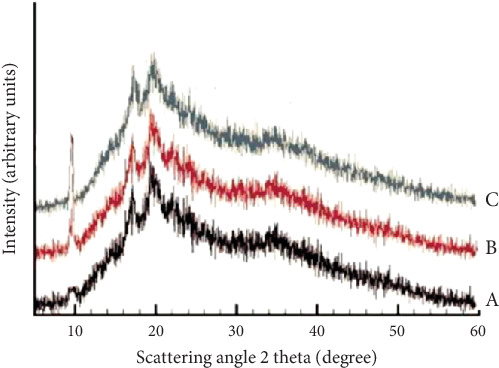
XRD graphs of treatments: (A) HPMC film, (B) HPMC-based film containing 5 wt% nanoclay, and (C) HPMC film containing 4.26 wt% nanoclay and 1.70% vol/vol DEO.

Figure 11 shows the XRD spectra of the HPMC-based films. The spectrum of the HPMC film shows two distinct peaks at 2*θ* of 17° and 20°. In this spectrum, a small peak is seen at 2*θ* 36°, which is related to the crystal structure of HPMC. Also, a very small peak at 2*θ* 10° is related to HPMC. Examination of the spectra related to (A) HPMC film, (B) HPMC-based film containing 5?wt% nanoclay, and (C) HPMC film containing 4.26?wt% nanoclay and 1.70% vol/vol DEO shows that these spectra are not much different from the spectrum related to HPMC, and only the peak intensities are slightly reduced. Therefore, it can be said that the addition of encapsulated essential oils and nanoclay and DEO nanoparticles has slightly reduced the crystal structure of the HPMC film. The reason for the decrease in the crystalline property of the film in the presence of encapsulated essential oils is probably due to the fact that the essential oils are essentially noncrystalline and can reduce the setting and order of polymer crystals by penetrating into the HPMC polymer chain. HPMC is the essential oil capsule wall and can reduce the crystal structure of the film. In other words, nanoclay and DEO nanoparticles were coated in small amounts by the film components, and no peaks related to these particles were observed. Saha et al. [44] studied pectin composite films containing nanoparticles and studied the effect of nanoparticles on the crystalline properties of the film. They reported that nanoparticles and composite components affect the crystal structure of the film, which confirms the XRD result of the present study [44].

### 3.8. Study of Controlled Release of Essential Oil From the Film

**Figure 12:**
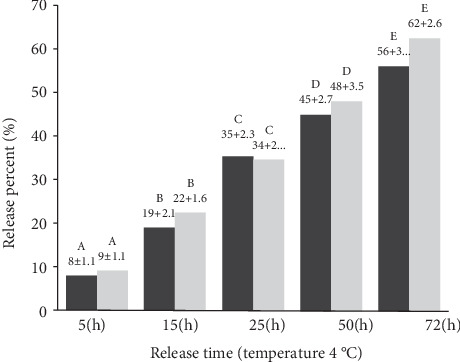
Release graphs of treatments: 1: HPMC-based film containing 5 wt% nanoclay and 2: HPMC film containing 4.26 wt% nanoclay and 1.70% vol/vol DEO. Similar letters on each bar indicate no significant difference (*p* > 0.05).

Figure 12 shows a controlled release diagram of the essential oil from the film at four into the simulant solution. As it is seen, the release of essential oil from the film into the environment occurs gradually and in a controlled manner. Release from the HPMC film containing 4.26 wt% nanoclay and 1.70% vol/vol DEO film occurs more frequently than in the HPMC-based film containing 5 wt% nanoclay film. It can be said that nanoclay and DEO nanoparticles affect the wall of the essential oil capsule and make the capsule wall more permeable, so the essential oil is more easily released into the environment. Temperature does not have a significant effect on the release rate of essential oil, which could be due to the fact that the capsule wall and the internal structure of the film are not affected by ambient temperature. Assadpour et al. [45] used the pectin/whey protein wall for encapsulation of folic acid and examined its release over time and reported that folic acid release occurs gradually and in a controlled manner. The results of the present study are consistent with their results.

### 3.9. The Effect of Coverings on Egg Quality

**Figure 13:**
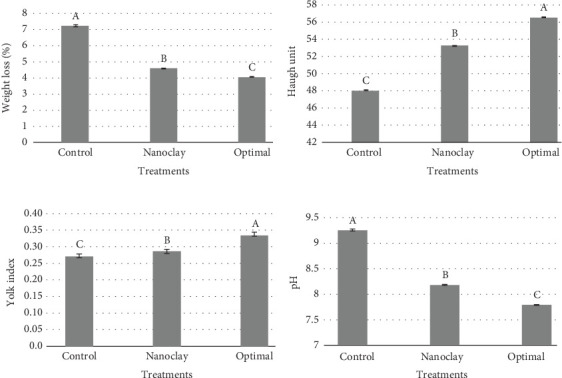
Comparison of egg quality parameters comprising (a) weight loss, (b) Haugh unit, (c) yolk index, and (d) pH of treatments of eggs coated by HPMC (control), HPMC containing 5 wt% nanoclay (nanoclay), and HPMC containing 4.26 wt% nanoclay and 1.70% vol/vol DEO (optimal) after a 4-week storage period at 20°C. Different letters on each bar indicate significant differences at *p* < 0.05.

**Figure 14:**
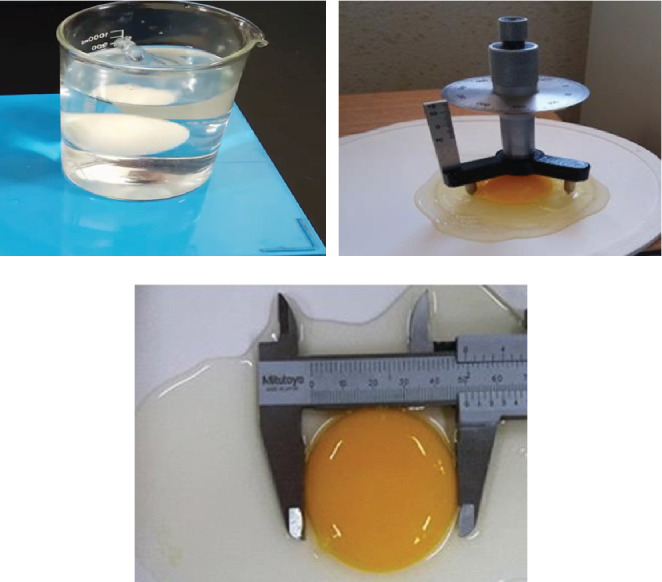
Image related to checking the quality of coated eggs. (a) Fresh egg test and (b, c) yolk width.

The study investigated the qualitative and sensory properties of eggs covered with three different films: an optimized formulation film with nanoclay and DEO, a film with nanoclay only, and a control film without nanoclay or DEO. The optimized formulation film resulted in eggs with a color similar to that of a natural egg, a pleasant smell and taste due to the presence of DEO, and more excellent durability. The presence of nanoclay and DEO in the formulation helped maintain the quality of the egg, making the optimized formulation film the best option for egg covering [46, 47]. Figures 13, 13, 13, and 13 illustrate a comparison of egg quality parameters, including weight loss (percent), Haugh unit, yolk index, and pH value after 4 weeks among different treatments in this study; also, an image related to checking the quality of coated eggs is shown in Figure 14.

#### 3.9.1. Weight Loss

The evaluation of weight loss in eggs plays a vital role in appraising the freshness of eggs during their storage. The data presented in Figure 13 demonstrates the progressive weight loss of different treatments after a 4-week storage period. Weight loss declined significantly (*p* < 0.05) with an optimal coating containing 4.26 wt% nanoclay and 1.70% DEO. This weight loss is mainly attributed to water's evaporation and carbon dioxide emission through the eggshell pores. Storage conditions such as temperature, air movement, and humidity affect the magnitude of water loss. These factors influence prolonged storage, particularly at ambient temperature. Our results showed that the optimal treatment could decline weight loss of eggs coated by HPMC from 7% in the control treatment to 4%. This result was in line with the findings of Sun et al. [48], who reported a 4% reduction in total weight loss of eggs coated by beeswax and 1.5% vol/vol basil essential oil compared to those without coatings after 28 days of storage. Similar to our results, other investigators reported that the Ag/AgCl/TiO_2_ nanocomposite treatment had the lowest weight loss (5.44%) and the lowest pH change after 27 days of egg storage [28].

#### 3.9.2. Haugh Index

The optimized sample had the highest Haugh unit values, and the control sample had the lowest Haugh unit values. The Haugh unit depends on the egg's weight and the height of the thickest part of the albumen. This index varies between 20 and 100, and the higher it is, the better the quality of the egg white. The Haugh unit increased in the methylcellulose hydroxypropyl film (Figure 13), possibly due to the reduced empty space between the film structures by the added nanoclay and DEO [49].

In another study, DEO was added to the HPMC film, and its effect on the Haugh unit was examined. The results showed that increasing the amount of DEO also increased the Haugh unit. Furthermore, adding coriander essential oil to the film can create hydrogen bonds and other intermolecular forces in the film structure, which increases the stability of the film and reduces the empty space. As a result, the Haugh unit rises. Therefore, increasing the Haugh unit in HPMC film by adding nanoparticles and DEO is due to the reduction of empty space, the increase in the active surface area of the film, the barriers to water vapor transfer, and the creation of intermolecular bonds in the film structure [50]. Similarly, Pires et al. [51] coated egg samples with rice protein–based films containing various essential oils (1% vol/vol), including tea tree, copaíba, and thyme. They observed that all egg treatments coated with essential oils demonstrated a higher Haugh index during storage at 20°C for 6 weeks. The decrease in the Haugh unit value is generally caused by the proteolysis of ovomucin, the cleavage of disulfide bridges, or the interaction between *α* and *β* ovomucins. Over time, the enzymes in the egg white hydrolyze the amino acid chains, disrupting the protein structure and releasing bound water, resulting in the fluidization of the egg white and a decrease in viscosity [51]. Moreover, the decrementation of the Haugh unit was associated with the liquefaction of the dense albumen. Other studies also recommended the use of the coating in order to maintain the Haugh unit during the shelf life period of eggs [48, 52].

#### 3.9.3. Yolk Index

Figure 13 demonstrates the comparison among three coating treatments of eggs after a storage period of 28 days. It was observed that the optimal coating maintained the yolk index around 0.35, which was significantly higher than that of HPMC film and HPMC/nanoclay (*p* < 0.05). The yolk index positively correlates with yolk quality, indicating that the coating constituting the essential oil and nanoclay plays a beneficial role in preserving yolk quality. Coatings effectively reduce the transfer of water and CO_2_ from the egg white through the shell during long-term storage [48, 51, 53, 54]. The increase in yolk width is due to water diffusion through the vitelline membrane from the egg white, which prevents egg white liquefaction and water absorption by the yolk, thereby minimizing the deterioration of yolk quality (Figure 14) [54].

#### 3.9.4. pH Measurement

The pH value of egg white indicates the level of acidity in the egg white and is of great importance to the egg industry. Many microorganisms require a slightly acidic environment, so the pH value of the egg white can be used as one of the critical factors in preventing the growth of harmful bacteria during egg storage [55]. The use of HPMC film may affect eggs' storage conditions, causing temperature and humidity changes. These changes can lead to a decrease in the pH value of the egg white [56]. Figure 13 showed that the control treatment had the highest pH value (9.21), and using nanocomposites could maintain a pH value of around 8. The best treatment was the optimal coating containing 4.26 wt% nanoclay and 1.70% DEO with a pH value of 7.8. In line with the latest findings, Pires et al. [51] reported a pH increment from 8.2 to 9.1 after a 4-week storage period in eggs coated by beeswax. Notably, incorporating 1% basil essential oil into beeswax could maintain a pH of around 8.6 after the same storage period [48].

## 4. Conclusion

Films and coverings play a crucial role in preserving food quality and extending shelf life in the food packaging sector. A recent study investigated the use of nanoparticles and essential oils in HPMC films, revealing significant improvements in microbial quality. The research team employed RSM and Design Expert software to precisely fine-tune the film formulation, taking into account specific amounts of nanoparticles and DEOs. Notably, the study results demonstrated a substantial impact of these input variables on key film properties, such as WVP and TS. The optimized model, adjusted with 4.26 wt% of nanoparticles and 1.70 vol% of dill essence, showed promising outcomes. Compared to other HPMC films, including those containing nanoparticles and a control sample, the optimized film exhibited superior antioxidant and antimicrobial activity. Subsequently, the researchers applied the optimized HPMC film to coat raw eggs and carefully assessed their quality characteristics. The results were compelling, showing minimal weight loss in the coated eggs, reduced egg white pH, and the highest yolk index and Haugh unit compared to other samples. These findings underscore the potential of optimized films in enhancing food preservation and quality.

## Figures and Tables

**Table 1:** Treatments investigated in the research.

**Number**	**Amount**
Treatment1	Control: HPMC film
Treatment1	Nanoclay: HPMC-based film containing 5 wt% nanoclay
Treatment1	Optimal: HPMC film containing 4.26 wt% nanoclay and 1.70% vol/vol DEO

**Table 2:** Independent variables used in modeling.

**Factors**	**Name**	**Unit**	**Type**	**Min**	**Max**	**Coded low**	**Coded high**	**Mean**	**Std. dev.**
*X*_1_	Nanoclay	(*V*/*V*) %	Numeric	0.00	5.00	−1 ↔ 0.73	+1 ↔ 4.27	2.50	1.44
*X*_2_	Dill essential oil	%(w)	Numeric	0.00	2.00	−1 ↔ 0.29	+1 ↔ 1.71	1.00	0.57

**Table 3:** The central composite design employed for the formulation of edible coating composition.

**Run**	**Space type**	**Factor 1**	**Factor 2**	**Response 1**	**Response 2**	**Response 3**
		**X**_1_ **: Nanoclay (%)**	**X**_2_ **: Dill essential oil (%)**	**WVP ((g.s** ^**−1**^ **.m** ^**−1**^ **.Pa** ^**−1**^ **)**	**TS (MPa)**	**EB (%)**
1	Center	2.5	1	1.74	23.09	24.41
2	Axial	5	1	1.55	24	22.3
3	Axial	0	1	2.31	16.21	30.05
4	Axial	2.5	2	1.57	18.63	27.65
5	Center	2.5	1	1.75	21.11	25.4
6	Factorial	0.73	1.70	1.65	17.45	29.24
7	Center	2.5	1	1.65	22.41	23.5
8	Axial	2.5	0	2.32	28.2	17.02
9	Center	2.5	1	1.76	21.17	24.46
10	Center	2.5	1	1.64	22.55	25.81
11	Factorial	4.26	1.70	1.49	20.08	26.08
12	Factorial	4.26	0.29	1.9	30.1	14.56
13	Factorial	0.73	0.29	2.31	26.6	22.31

**Table 4:** ANOVA study for the model fitting.

**Parameter**	**Sum of squares**	**df**	**Mean squares**	**F** **-value**	**p** ** value**	
*WVP (g.s^−1^.m^−1^.Pa^−1^)*						
Model	1.02	5	0.2041	21.87	> 0.0004	Significant
*X*_1_—Nanoclay	0.338	1	0.338	36.23	0.0005	
*X*_2_—Dill essential oil	0.567	1	0.567	60.79	0.0001	
*X*_1_ *X*_2_	0.015	1	0.015	1.67	0.236	
*X*_1_	0.051	1	0.051	5.51	0.05	
*X*_2_	0.060	1	0.060	6.51	0.038	
Lack of fit	0.519	3	0.017	5.13	0.074	Not significant
*TS (MPa)*						
Model	170.45	2	85.22	20.63	> 0.0001	Significant
*X*_1_—Nanoclay	36.75	1	36.75	12.78	0.005	
*X*_2_—Dill essential oil	133.69	1	133.69	46.86	> 0.0001	
Lack of fit	26.64	6	4.27	5.48	0.060	Not significant
*EB (%)*						
Model	223.43	5	44.69	40.74	> 0.0001	Significant
*X*_1_—Nanoclay	59.79	1	59.79	54.51	0.0002	
**X**_2_—Dill essential oil	140.14	1	140.14	127.76	> 0.0001	
*X*_1_ *X*_2_	5.27	2	5.27	4.80	0.064	
*X*_1_^2^	1.27	1	1.27	1.16	0.317	
*X*_2_^2^	15.49	1	15.49	14.12	0.007	
Lack of fit	4.38	3	1.46	1.77	0.292	Not significant

**Table 5:** The fit coefficients of the proposed models in the Design Expert software.

	**Std. deviation**	**Mean**	**CV (%)**	**R** ^2^	**R** ^2^_**A** **d** **j** **u** **s** **t** **e** **d**_	**R** ^2^_**P** **r** **e** **d**_.
WVP (g.s^−1^.m^−1^.Pa^−1^)	0.09	1.82	5.31	0.939	0.896	0.641
EB (%)	1.7	22.43	7.56	0.855	0.826	0.700
TS (MPa)	1.05	24.06	4.35	0.966	0.943	0.843

## Data Availability

The data that support the findings of this study are available from the corresponding author upon reasonable request.
